# Selective serotonin reuptake inhibitor use associates with apathy among depressed elderly: a case-control study

**DOI:** 10.1186/1744-859X-6-7

**Published:** 2007-02-21

**Authors:** Nahathai Wongpakaran, Robert van Reekum, Tinakon Wongpakaran, Diana Clarke

**Affiliations:** 1 Department of Psychiatry, Division of Geriatric Psychiatry, Faculty of Medicine, University of Toronto. Baycrest. Canada; 2 Department of Psychiatry, Faculty of Medicine, University of Toronto. Baycrest. Canada; 3 Department of Psychiatry, Faculty of Medicine, University of Toronto. Canada; 4 Kunin-Lunenfeld Applied Research Unit, Baycrest. Canada

## Abstract

**Background:**

It has been reported for over the past decade that the use of selective serotonin reuptake inhibitors (SSRI's) may associate with the emergence of apathy. The authors hypothesized that depressed patients treated with SSRI's would show more signs of apathy than patients treated with non-SSRI antidepressants. This case control study was conducted to investigate the possibility of the association between SSRI use and the occurrence of apathy.

**Methods:**

Baycrest Centre for Geriatric Care's Day Hospital Database of elderly depressed patients who received antidepressants was divided into 2 groups depending on antidepressant use at discharge: SSRI user group-SUG, and non-SSRI user group-NSUG. Apathy scales developed by the authors were selected from the Geriatric depression Scale (GDS) and the Hamilton Rating Scale for Depression (HAMD), and were titled as GDS-apathy subscale (GAS) and HAMD-apathy subscale (HAS). Demographic data, baseline apathy, underlying medical conditions and medication use were studied. Proportion, analysis of variances, Chi-square test, odds ratio with 95% confidence interval were reported.

**Results:**

Among 384 patients (160 SUG and 224 NSUG), mean GDS and HAM-D at discharge were 12.46 and 10.61 in SUG, and were 11.37 and 9.30 in NSUG, respectively. Using GAS for apathy assessment, 83.7% of patients in SUG and 73.4% in NSUG stayed apathetic at discharge. As evaluated by HAS, 44.2% of patients in SUG and 36.5% in NSUG stayed apathetic. SSRI use was not a predictor of apathy at admission, while it was at discharge, p = 0.029. The SUG showed more patients with apathy than that found in NSUG (adjusted OR = 1.90 (1.14–3.17). Age 70–75 years tended to be a predictor for the apathy (p = 0.058). Using HAS, age 70–75 years and living situation were associated with apathy at discharge, p = 0.032 and 0.038 respectively.

**Conclusion:**

Even though depression was improved in elderly patients receiving antidepressants, apathy appeared to be greater in patients who were treated with SSRI than that found in patients who were not. Frontal lobe dysfunction due to alteration of serotonin is considered to be one of the possibilities.

## Background

Apathy is a common behavioral syndrome characterized as a decrease in (or lack of) interest, motivation, or initiation of action [1,2]. Apathy can be found among patients with depression, psychosis, dementia, traumatic brain injuries, etc [1]. The syndrome is associated with poor functioning, poor illness outcome, and a negative impact on caregivers. Although apathy and depression are related, the two syndromes are distinct from each other [1,3,4].

Selective serotonin reuptake inhibitors (SSRI's) are widely used in treating depressive and anxiety disorders in elderly persons. Over the past decade, four case reports revealed 12 cases, receiving various SSRI's, who developed apathy, amotivation or a frontal lobe syndrome [5-8]. However, all of these cases were adults or adolescents.

Frontal-subcortical dysfunction is proposed as a cause of apathy and depression [1,2,9]. In frontal areas, there is a counterbalance between serotonergic and adrenergic function. Two randomized controlled trials, comparing SSRI's and selective noradrenaline reuptake inhibitor (NARI) in depression, reported that serotonergic manipulation shows less improvement in motivation, at the group level of analysis, than do noradrenergic agents, even though depressive symptoms are improved [10,11].

The effect of SSRI's exposure on the risk for apathy has not been well studied. We conducted a case control study to evaluate the risk of apathy among elderly depressed day-hospitalized patients treated with, or without, SSRI's. The study utilized an existing database, which has recorded clinical data, at admission and discharge, from patients treated in the Day Hospital for Depression at Baycrest.

The authors hypothesized that depressed patients treated with SSRI's would show more signs of the apathy syndrome at discharge from the Day Hospital, than patients treated with non-SSRI antidepressants. Therefore, the purpose of this study was to investigate the possible association between SSRI use and the apathy syndrome in an elderly depressed group treated in day hospital setting.

## Methods

Information related to all depressed elderly given an antidepressant in the Day Hospital from April 1986 to January 2005 was identified in the database. Apathetic and non-apathetic groups were defined on the basis of data recorded at discharge from day hospital (see below). The authors also divided the patients into 2 groups depending on information regarding antidepressant used at discharge: 1) SSRI's user group (SUG) and 2) Non-SSRI's user group (NSUG).

Data from first admission generally included demographic and clinical characteristics of the study sample, and data related to potential confounding variables. Scales representing apathy were extracted from the primary scales used for assessing the patients at admission (to control for baseline apathy) and were compared between both groups at discharge. These scales utilized items from the Geriatric Depression Scale (GDS) [12] and the 21-item Hamilton Rating Scale for Depression (HAMD-21) [13]. Items selected (see below) were chosen by consensus of the principal investigator and two local experts on the Apathy Syndrome (Drs. Tiffany Chow and Robert van Reekum).

### Study population

Study population included individuals aged 55 and older who had been diagnosed as depressed and who received any type of antidepressant while in the Day Hospital. Patients who did not receive pharmacological therapy were excluded from the study. In order to avoid potential biases, in the case of multiple admissions, only the data from the first admission was used.

### Scales for apathy

Scales for directly assessing apathy were not included in the database. Thus, the investigators retrieved some associated items from the GDS and the HAMD-21 for apathy evaluation.

From the GDS, the following items were used: item 2 (Have you dropped many of your activities and interests?), item 12 (Do you prefer to stay at home, rather than going out and doing new things?), and item 20 (Is it hard for you to get started on new projects?). The extracted scale from the GDS was titled the GDS-apathy subscale (GAS); scores range from 0 to 3. A score of '0' represented 'non-apathy', and scores from 1–3 were grouped as 'apathy' in this study.

From HAMD-21, item 7 was retrieved. The question is about 'Work and activities'; score of 0 = No difficulty; 1 = Incapacity, fatique or weakness related to activites, work or hobbies; 2 = Loss of interest in activites, hobbies or work-reported by patient or listlessness, indecision and vacillation (has to push self); 3 = Decrease in actual time spent in activities or decrease in productivity; 4 = Patient engages in no activity or fails to perform unassisted). The item was titled HAMD-apathy subscale (HAS); scores range from 0–4. Scores of 0–1 on this item were defined as 'non-apathy' per se, and scores from 2–4 were grouped as 'apathy'.

### Potential confounders

Age, gender, baseline apathy (per the scale developed by the authors), primary language used, living situation, underlying medical conditions, smoking, and marijuana (or other substance use) might all be possible confounding factors [2,14]. Not only antidepressants are used amongst patients in Day Hospital, but also other medications. Thus, information on the use of other drugs that might induce apathy through their pharmacological action was also studied. These drugs might include sedative-hypnotic drugs, sedating drugs, anticholinergic drugs, antiepileptic drugs, antipsychotic drugs, etc.

For all medication use, only the name of the medication was recorded in the database. Data regarding dosage, date started and discontinued, etc was not available.

Underlying or co-morbid medical conditions which might be confounders include: 1) diseases of the central nervous system such as Alzheimer's disease, cerebrovascular diseases, Parkinson's disease, etc., 2) endocrine disorders such as hyperthyroidism, hypothyroidism, panhypopituitarism, and 3) nutritional diseases such as vitamin deficiency.

### Statistical analysis

Demographic data (such as gender, marital status, living situation, etc.) and diagnoses were reported by proportion. Age and duration of stay were calculated by mean. In order to compare means of age, gender, length of stay, education, marital status, living status, primary language used, total GDS, total HAMD-21, GAS and HAS at both admission and discharge in SUG and NSUG, an analysis of variance (ANOVA) was applied. A Chi square test was calculated for analyzing the differences among comorbid diseases and medication use between the two groups. Multivariate logistic regression analyses were conducted for analyzing the apathy risk, and for assessing the predictive variables. Odds ratio (OR) with 95% confidence interval was calculated for the apathy syndrome between SUG and NSUG.

## Results

The first admission data from 824 elderly depressed patients were received from the database. Six hundred (600) cases received antidepressants. Three hundred and eighty four cases had complete GDS for both admission and discharge and were included in this study.

With respect to Axis I diagnosis, 249 patients (64.8%) had been diagnosed with major depressive disorder, 18.2% had major depressive disorder and dysthymia (or 'double depression'), 5.7% had dysthymia, 5.2% were patients with depressive disorder due to a general medical condition, 4.7% were bipolar depressed patients, and 1.3% had adjustment disorder with depressed mood.

Some patients had comorbid psychiatric disorders; 63 cases had anxiety disorders or other neuroses, 29 patients suffered from substance related disorders, and 12 cases had psychotic disorders.

The demographic data, scores for depression, selected apathy scores, co-morbid or underlying axis III diseases and other medication use during the stay are indicated in Table 1. Due to missing HAMD, the data related to the scale were available in 290 patients.

**Table 1:** Comparison of variables between SUG and NSUG

***Variables***	***SUG (n = 160)***	***NSUG (n = 224)***	***Remarks***
**Demographic data**			
Mean age (years)	74.6 ± 6.9	75.7 ± 6.9	
%Female	72.5	72.3	
%Marital status (non-married)	64.4	57.1	
%Primary language use (non-English)	56.3	44.2	
%Living status (alone)	45.6	48.2	
%Education (< high school)	51.9	49.1	
Average length of stay (days)	136.7 ± 36.0	137.3 ± 38.1	
**Scales**			
Mean HAS at admission	1.98 ± 0.98	2.08 ± 0.98	a
Mean HAS at discharge	1.13 ± 0.89	0.99 ± 0.89	a
Mean total HAM-D 21 at admission	18.57 ± 6.33	18.67 ± 6.27	a
Mean total HAM-D 21 at discharge	10.61 ± 6.48	9.30 ± 6.15	a, p = 0.046
Mean GAS at admission	2.18 ± 0.88	2.13 ± 0.83	
Mean GAS at discharge	1.43 ± 1.01	1.31 ± 1.05	
Mean total GDS at admission	19.34 ± 6.23	18.75 ± 6.11	
Mean total GDS at discharge	12.46 ± 7.45	11.37 ± 6.79	
**Co-morbid axis III illness**			
Dementia	5	12	
Parkinson's disease	3	8	
Stroke	21	29	
Head injury	6	5	
Hypertension	60	87	
DM	27	28	
Hyperthyroidism	1	3	
Hypothyroidism	26	29	
**Medication use**			
Benzodiazepines	89	128	
Hypnotics	144	207	
Antipsychotics	18	26	

a; n = 290, nSUG = 108, nNSUG = 182

The number of patients on SSRI's was 160, and 224 received non-SSRI antidepressants, i.e. heterocyclic antidepressants (HCAs), mono-amine oxidase inhibitors (MAOIs), serotonin and noradrenaline reuptake inhibitor (SNRI), noradrenergic and specific serotonergic antidepressant (NaSSA), serotonin reuptake and 5HTs inhibitor, and atypical noradrenaline and dopamine reuptake inhibitor. The SSRI's including in descending order of frequency of use: sertraline, paroxetine, fluoxetine, citalopram, and fluvoxamine.

ANOVAs revealed no significant differences between SUG and NSUG with respect to age, gender, length of stay, level of education, marital status, living status, primary language used, mean HAMD-21 at admission, or mean GAS, GDS, HAS at both admission and discharge (p > 0.05). Mean HAMD-21 at discharge in SUG was significantly greater than that in NSUG (p = 0.046).

In SUG, 153 patients were apathetic (by GAS) at admission, and 128 patients remained apathetic at discharge. Two hundred and fourteen patients in NSUG were apathetic at admission, and 157 patients remained apathetic at discharge. From this data, prevalence of apathy at admission using GAS was 95.6%, while at discharge, it was 74.2%. As presented in Table 2, only 290 patients had completed HAS. In terms of apathy at discharge, as assessed by the HAS, 34 patients (out of 108) in SUG, and 50 patients (out of 182) in NSUG, remained apathetic.

**Table 2:** Number of patients with apathy upon antidepressant use

***Antidepressants***	***Apathy using GAS (n = 384)***			***Apathy using HAS (n = 290)***		
	**At admission**		**OR**	**At admission**		**OR**
	**Yes**	**No**	**(95% CI)**	**Yes**	**No**	**(95% CI)**
SUG	153	7	1.02 b (0.38–2.74)	77	31	0.82 (0.48–1.39)
NSUG	214	10		137	45	
	**At discharge**			**At discharge**		
	**Yes**	**No**		**Yes**	**No**	
SUG	128	32	1.71 (*) (1.06–2.76)	34	74	1.21 (0.72–2.04)
NSUG	157	67		50	132	

Odds Ratio are presented as crude ratio.

(*) The adjusted OR = 1.90 (1.14–3.17)

With regard to the evaluation of the association between apathy using GAS at admission and all possible variables, the authors found that demographic data, co-morbid Axis III diseases and medication used, including SSRI's (p = 0.961), were not predictive factors for apathy at admission (all p > 0.05). The crude OR was 1.02 (0.38–2.74).

Regarding apathy at discharge using GAS, the length of stay (p = 0.011) was one of the predictor variables for apathy. The number of people with apathy who were admitted for between 3 and 6 months was less than that in the groups with shorter or longer stay (p at 3, 4, 5 and 6 months were 0.015, 0.002, 0.008 and 0.045 respectively). Moreover, age group of 70–75 years tended to be a predictor for apathy (p = 0.058). The number of people with apathy in this age group tended to be less than in other groups. Apathy was not related to either co-morbid axis III diseases or to non-antidepressant medication use (all p > 0.05). SSRI use was one of the predictors for apathy (p = 0.029). The SUG showed more patients with apathy than in NSUG with a crude OR of 1.71 (1.06–2.76 95% CI) and an adjusted OR of 1.90 (1.14–3.17 95% CI).

In the evaluation of apathy among the 290 patients who had complete and valid HAMD-21 data, the authors found no evidence of predictor variables for apathy at admission. The variables predicting apathy at discharge were: age group of 70–75 years (p = 0.032) and living situation (p = 0.038). SSRI use was not a predictor for apathy at discharge using the HAS (p = 0.045) with a crude OR of 0.82 (0.49–1.39).

## Discussion

The authors acknowledge that the study is limited by the measurement of apathy used, as it was derived from depression scales, and has not been validated (beyond content validation from local experts). The study is, of course, also limited by the data contained in the database (e.g. dose of medications not available, duration of use not available, etc). Despite these limitations, important relationships between apathy and potential contributors to apathy (e.g. SSRI use) were found.

In both SUG and NSUG, all apathy scores at discharge were less than at admission. Therefore, both SSRI's and non-SSRI's appeared to be efficacious in treating the apathy of depression. Even though there was no difference between apathy scores as measured by GAS and HAS between both groups, the difference in the number of cases who remained apathetic at discharge between groups was different.

Apathy at admission, using both selected scales, did not have strong relationships with any of the possible predictor variables.

Apathy at discharge was predicted by SSRI use, as indicated by the OR of 1.91 in the SUG (as assessed by the GAS). To discuss the hypothesis that apathy syndrome is caused by SSRI use, the criteria of Sir Bradford Hill [15] will be briefly considered. The 9 criteria are *1) the strength of the association, 2) the consistency of the association, 3) biologic plausibility, 4) the temporal relationship, 5) the biologic gradient, 6) its specificity, 7) coherence, 8) experimental evidence, and 9) analogy*. However, van Reekum et al. [16] have proposed that for assessing causation in neuropsychiatry, criteria 1 to 4 are the most relevant.

SSRI use was shown, in this study, to be associated with apathy. This is consistent with the previous reports [3,5-8,10,11], in spite of the different settings and age group of the participants. It is biologically possible that frontal lobe dysfunction, induced by SSRI's, may be responsible for the apathy seen in the SUG in this study. There are several counterbalances of neurotransmitters in the brain. With respect to a counterbalance of serotonin and dopamine, Kapur et al. [17] proposed that prolonged and excessive serotonin in the synapse may lead to a decrease in transmission of dopamine in the frontal lobe. A decrease of dopamine is one of the potential causes of the apathy syndrome as with the apathy seen in people with Parkinsonism. Additionally, Golomb et al. [18] stated that depression is associated with both low serotonin and high acetylcholine function. High serotonin may cause a decrease in acetylcholine, and vice versa, which can cause an increase in dopamine function thereafter. The relationship between serotonin and noradrenaline is another possible mechanism [10,11]. Desensitization of postsynaptic 5-hydroxytryptamine (5-HT) receptors is a recent finding resulting in rebound symptoms in prolonged use of paroxetine [19]. At present, we do not yet have enough data to know how altering serotonergic functioning might cause apathy.

In terms of the temporal relationship between SSRI use and apathy, this study is limited, as the database lacked information on start date, and duration of the use of medication.

Length of stay seemed to have a relationship with apathy. The direction of causation is unclear; prolonged day hospital stay might have caused apathy, or, perhaps more likely, apathy caused patients, families, and the day hospital team to consider longer day hospital stays.

Further research, using prospective data (e.g. medication use), and better validated apathy scales, in large sample sizes (such as population-based or national surveys) is supported by the results of this study. The investigation of the effect on apathy of SSRI users in all age groups will also be required. Patient education, related to the potential for SSRI's to cause apathy, should be considered.

## Conclusion

Apathy at discharge appeared to be greater in elderly depressed patients who were treated with SSRI's than that found in patients were not. Further studies with prospective design are required. Patients and caregivers should be informed to be more aware of this potential adverse effect when using SSRI's. Careful monitoring for apathy, and consideration of switching antidepressant class in patients presenting with apathy, should be undertaken in all patients receiving an SSRI.

## Competing interests

The author(s) declare that there is no competing interest.

## Authors' contributions

NW conceived, designed the study, performed the statistical analysis and drafted the manuscript. RvR designed the study, helped with statistical analysis, and corrected the manuscript. TW participated in data management and statistic analysis. DC helped with the database collection and statistic analysis. All authors read and approved the final manuscript.
